# Multicenter study on caries risk assessment in adults using survival Classification and Regression Trees

**DOI:** 10.1038/srep29190

**Published:** 2016-07-06

**Authors:** Masumi Arino, Ataru Ito, Shozo Fujiki, Seiichi Sugiyama, Mikako Hayashi

**Affiliations:** 1 Department of Restorative Dentistry and Endodontology, Osaka University Graduate School of Dentistry, 1-8 Yamadaoka, Suita, 565-0871, Japan; 2 The Japan Health Care Dental Association, 1-45-15 Sekiguchi, Bunkyo-ku, Tokyo 112-0014, Japan

## Abstract

Dental caries is an important public health problem worldwide. This study aims to prove how preventive therapies reduce the onset of caries in adult patients, and to identify patients with high or low risk of caries by using Classification and Regression Trees based survival analysis (survival CART). A clinical data set of 732 patients aged 20 to 64 years in nine Japanese general practices was analyzed with the following parameters: age, DMFT, number of *mutans streptococci* (SM) and *Lactobacilli* (LB), secretion rate and buffer capacity of saliva, and compliance with a preventive program. Results showed the incidence of primary carious lesion was affected by SM, LB and compliance with a preventive program; secondary carious lesion was affected by DMFT, SM and LB. Survival CART identified high-risk patients for primary carious lesion according to their poor compliance with a preventive program and SM (≥10^6^ CFU/ml) with a hazard ratio of 3.66 (*p *= 0.0002). In the case of secondary caries, patients with LB (≥10^5^ CFU/ml) and DMFT (>15) were identified as high risk with a hazard ratio of 3.50 (*p* < 0.0001). We conclude that preventive programs can be effective in limiting the incidence of primary carious lesion.

Dental caries has been an important public health problem worldwide. In modern caries management has shifted from the concept of “drilling and filling” to caries risk assessment^1^. Proper assessment of an individual’s risk is as important as precise diagnosis of carious lesions^2^. Information about an individual’s caries risk and state of carious lesions should be used in making a personal treatment plan. This concept of patient-centered caries management is well-accepted worldwide. However, in most cases, and especially in adults, regular check-ups are recommended to control caries without proper risk assessment^3^,^4^,^5^. Most research on caries risk assessment has focused on children^6^,^7^; much less is known about the effectiveness of preventive dental therapies in controlling caries in adults^8^,^9^,^10^,^11^. This is mainly because collecting appropriate clinical data relevant to an individual adult patient is often problematic. Many adult patients already have multiple restorations and may have ongoing treatment programs. In these circumstances, assessing their vulnerability to caries can be difficult, because the quality of treatment directly affects an individual’s risk.

In our previous studies, we had the advantage of being able to access relatively complete dental treatment records over a 15-year period of adult patients whose saliva and cariogenic bacteria had been tested before they started regular preventive therapy^10^,^11^. Equally important, the patients received initial restorative and periodontal treatment before testing so as to eliminate possible negative effects of inferior oral conditions. We successfully identified patients with a high or low risk of primary caries by using Classification and Regression Trees (CART) according to the number of specific cariogenic bacteria, as well as those at risk of secondary caries according to the number of particular bacteria and previous caries experience^10^. We also proved that the value of these bacteria as markers changed after three years of a preventive program^11^. These findings are clinically meaningful, but their usefulness is limited by being confined to a single clinic. Their validity should be clarified by conducting similar analysis using multicenter data.

CART based survival analysis (survival CART) is a sophisticated data mining method using a combination of CART and survival curves^12^,^13^. Survival CART enables identification of patients with high and low risk of caries using significant clinical parameters with specific thresholds. It also provides information how long it takes to develop a new carious lesion for each high and low risk group. This information helps in planning a personal regular preventive program with an interval appropriate to the individual’s risk of caries.

The purpose of this retrospective observational cohort study was to investigate the effectiveness of preventive therapy in reducing the incidence of primary and secondary carious lesion in adult patients. We also used survival CART to investigate significant risk factors correlated with the onset of new carious lesions in patients receiving long-term preventive therapy.

## Results

### Patient characteristics

**Figure 1:**
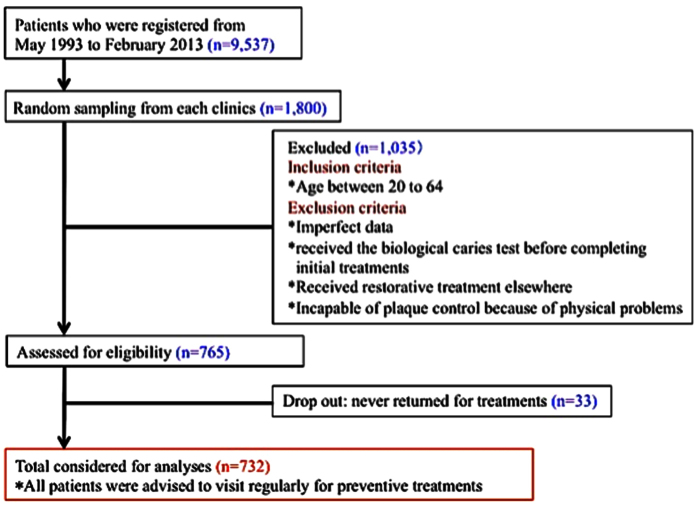
The selection process for patients undergoing survival and survival CART analysis.

The patient selection method for this study is summarized in Fig. 1. Although the data of 1,800 patients were selected from nine dental practices, only 732 patients were used for CART analysis. The main reasons for excluding the others were that 127 patients were out of the age range of 20 to 64 years; 764 patients underwent bacteria and saliva testing before completing the initial restorative and periodontal treatments; 144 had imperfect data. Initially, 765 patients underwent bacteria and saliva testing when completing their initial treatments and were advised to undergo regular preventive treatment. However, 33 patients dropped out of the preventive program. Finally, 732 patients (224 male and 508 female) with an average age of 42.2 ± 12.5years were eligible.

Typically for Japan, the dental practices were run by a single dentist with three to seven hygienists. Almost all patients used the government health insurance scheme, which has near-universal coverage and covers most common procedures. There were no major ethnic, demographic, economic, or social distinctions among patients in what is “the most middle-class nation on earth”. The preponderance of female patients is because men generally receive dental treatment closer to their work place, and sometimes in clinics provided by their employers.

The average DMFT at the time of caries risk assessment was 15.6 ± 6.4. The average saliva secretion rate was 7.7 ± 4.0 ml. A high saliva buffer capacity was found in 67.5% of the patients, a moderate capacity was found in 21.7%, and a low capacity was found in 10.8%. In terms of compliance with the prevention program, 63.1% of patients exhibited good compliance while 36.9% exhibited poor compliance. Approximately 63% of patients showed moderate levels of SM (1 × 10^5^–5 × 10^5^ CFU/ml) and the rest were categorized as low (<1 × 10^5^ CFU/ml, 15%) or high (≥1 × 10^6^ CFU/ml, 22%) levels (Appendix 1). More than 90% of the patients had low (<1 × 10^3^ CFU/ml) to moderate (<1 × 10^5^ CFU/ml) levels of LB, while less than 10% had high levels (≥1 × 10^6^ CFU/ml). In three years, 72 (9.8%) patients developed primary carious lesions and 89 (12.2%) developed secondary carious lesions.

### Survival analysis of primary and secondary caries lesion

**Figure 2:**
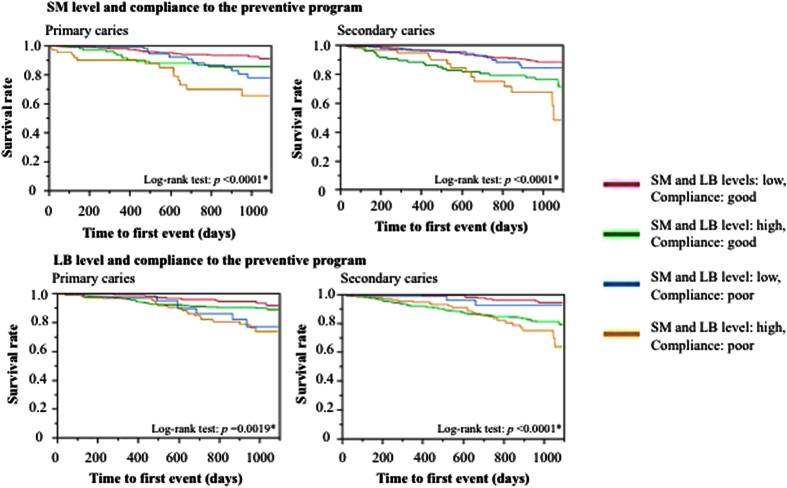
Survival analyses for the incidence of primary and secondary caries, focused on a combination of specific cariogenic bacteria level and compliance with the preventive program using the Kaplan–Meier and log-rank tests. SM level: high (higher than 1 × 10^6^ CFU/ml); low (1 × 10^6^ CFU/ml or lower). LB level: high (1 × 10^4^ CFU/ml or higher); low (lower than 1 × 10^4^ CFU/ml). Each patient’s compliance with the preventive program was categorized as follows: good (regular visits + sometimes delayed visits); poor (irregular visits + emergency visits only). *Statistically significant at a level of *p* < 0.05.

Single regression analysis showed that the period until the first outbreak of primary carious lesion was affected by SM (*p *= 0.0010), LB (*p *= 0.0027) and compliance with a preventive program (*p *= 0.0006); that of secondary carious lesion was affected by DMFT (*p* < 0.0001), SM (*p* < 0.0001) and LB (*p* < 0.0001) (Table 1). Figure 2 and Appendix 2 show the combination of effects of SM or LB and compliance with the preventive program on the development of new primary and secondary carious lesions. Patients complying with the preventive therapy infrequently developed new primary carious lesions, although their SM and LB levels were high. In contrast, patients with an SM level higher than 1 × 10^6^ CFU/ml or an LB level higher than 1 × 10^4^ CFU/ml were vulnerable to secondary caries, with hazard ratios of 2.41 (95%CI, 1.41–4.03; *p *= 0.0017) or 4.00 (95%CI, 2.02–9.11; *p* < 0.0001), regardless of whether they received regular preventive therapy. When SM and LB levels were lower than 1 × 10^6^ CFU/ml and 1 × 10^4^ CFU/ml, the onset of secondary carious lesion was not as frequent, even though patients did not fully comply with preventive therapy. As might be expected, patients with SM levels higher than 1 × 10^6^ CFU/ml and with irregular dental visits were vulnerable to both primary and secondary caries, with hazard ratios of 4.73 (95%CI, 2.40–8.90; *p* < 0.0001) and 3.63 (95%CI, 1.89–6.56; *p *= 0.0003), respectively. Patients with LB levels higher than 1 × 10^4^ CFU/ml who visited the dentist irregularly were vulnerable to primary and secondary caries, with hazard ratios of 3.75 (95%CI, 1.85–8.07; *p *= 0.0002) and 6.09 (95%CI, 2.84–14.5; *p* < 0.0001), respectively.

### Risk of onset of primary and secondary caries

**Figure 3:**
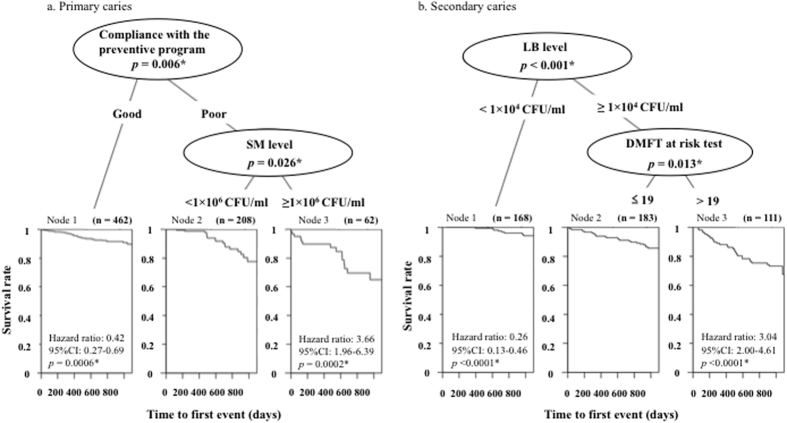
Survival CART for the incidence of primary and secondary caries within three years. For primary caries (**a**) three nodes were identified according to compliance with the preventive program and the SM level. Patients with good compliance were categorized as low-risk (Node 1) with a hazard ratio of 0.42; while patients with poor compliance and an SM level of ≥1 × 10^6^ CFU/ml were categorized as high-risk (Node 3) with a hazard ratio of 3.66. For secondary caries (**b**) three nodes were also identified according to the LB level and DMFT. Patients with an LB level of < 1 × 10^5^ CFU/ml were categorized as low-risk (Node 1) with a hazard ratio of 0.35. Patients with an LB level of ≥1 × 10^5^ CFU/ml and DMFT >15 were categorized as high-risk (Node 3) with a hazard ratio of 3.50. *Statistically significant at a level of *p* < 0.05.

Figure 3a shows the result of survival CART analysis for the onset of primary carious lesion. It demonstrates that patients’ relative risk of primary caries could be identified by compliance with the preventive program and the SM level. Patients with good compliance with the preventive program were categorized as the low risk with a hazard ratio of 0.42 (95%CI, 0.27–0.69; *p *= 0.0006); patients with poor compliance and an SM level higher than 1 × 10^6^ CFU/ml were categorized as the high risk with a hazard ratio of 3.66 (95%CI, 1.96–6.39; *p *= 0.0002). In the low-risk group (Node 1 in Fig. 3a), over 90% of patients did not develop new caries within three years; while 40% did in the high-risk group (Node 3 in Fig. 3a). The Survival rate in the high-risk group dropped sharply after 400 days.

Figure 3b shows the result of survival CART analysis for the onset of secondary carious lesion. Patients with a higher or lower risk for secondary caries were identified by their LB level and DMFT. Patients with an LB level lower than 1 × 10^5^ CFU/ml were categorized as the low risk with a hazard ratio of 0.35 (95%CI, 0.23–0.54; *p* < 0.0001). Patients with an LB level higher 1 × 10^5^ CFU/ml and DMFT greater than 15 were categorized as the high risk with a hazard ratio of 3.50 (95%CI, 2.30–5.31; *p* < 0.0001). In the low-risk group (Node 1 in Fig. 3b), over 90% of patients did not develop new caries within three years; approximately 30% did in the high-risk group, where the onset of secondary carious lesions steadily increased during the observation period (Node 3 in Fig. 3b).

## Discussion

The data of 1800 patients were selected from nine dental practices, but only 732 data were used for Survival CART analysis. We have checked the samples of 732 patients and confirmed that there were no biases in terms of the age, sex and DMFT from the 1800 patients originally selected. The main reasons for excluding the data of 1035 patients were 662 patients underwent biological caries testing before completing the initial restorative and periodontal treatments. This may be because clinicians use the biological caries test results to explain patients’ initial oral conditions and encourage them to control their plaque. However, when analyzing the inherent characteristics of the cariogenic bacteria of an individual, it is essential to conduct the biological caries testing after completing the initial treatments to avoid the possible negative effects of inferior oral conditions. Those patients who underwent caries testing before the initial treatments showed higher SM and LB levels than did the patients who underwent caries testing after the treatments; this is because the quantity and quality of cariogenic bacteria can be largely affected by untreated cavities and inferior restorations.

Cox regression analysis showed that SM and LB levels and compliance with the preventive program were significant factors in the development of primary carious lesion, while the DMFT, and SM and LB levels were significant for secondary carious lesion (Table 1). Because the SM and LB levels were identified as significant for outbreaks of both primary and secondary carious lesion, survival analysis was performed on SM and LB levels to clarify the effectiveness of preventive therapies (Fig. 2). These factors were also identified by survival CART analysis to categorize patients with higher and lower caries risk for primary and secondary caries (Fig. 3). The consistency among these results indicates the appropriateness of using survival CART analysis in helping to identify an individual’s caries risk.

In the previous study, we analyzed same clinical data set of 732 patients with CART analysis^14^. It showed that patients with an SM level higher than 1 × 10^6^ CFU/ml and poor compliance were categorized as the high-risk for primary carious lesion with an odds ratio of 3.08 (95%CI, 1.55–5.79; *p *= 0.0018), and that patients with an LB level higher 1 × 10^4^ CFU/ml and SM level higher than 1 × 10^6^ CFU/ml were categorized as the high-risk for secondary carious lesion with an odds ratio of 3.69 (95%CI, 2.29–5.91; *p* < 0.001). Comparing theses results with the results of Survival CART analysis, there were differences in the high-risk patients for secondary carious lesion. When we focused on only incidence of a secondary carious lesion during an observation period, cariogenic bacteria affected largely and the threshold of LB was low level with 1 × 10^4^ CFU/ml. However when considered the period until the first outbreak of secondary carious lesion, the threshold of LB was higher and picked up DMFT. This caries risk model with Survival CART can find the high-risk patients who will be vulnerable secondary carious lesion earlier within three years.

The results of survival analysis (Fig. 2) and survival CART (Fig. 3) indicated that preventive therapy was effective in limiting the incidence of primary carious lesion; on the other hand conventional preventive therapy alone cannot perfectly control the outbreak of secondary carious lesion. Clinically, these findings highlight the importance of preserving sound enamel surfaces and expanding the potential for remineralizing enamel infected with incipient caries. It is critically important to provide high-quality restorations with good marginal adaptation to prevent secondary caries. The essential message is to avoid conventional “drilling and filling” because multiple restorations increase the incidence of secondary caries.

The results of survival CART indicated that patients categorized as high risk for primary and secondary caries - whose survival curves steadily dropped (Fig. 2), needed to receive personalized intensive preventive care, such as enhanced plaque control^15^, applying topical fluoride^16^,^17^, use of high concentration fluoride toothpaste and mouth wash^18^,^19^,^20^, and improving their dietary habits^21^,^22^; low-risk patients only needed an annual check-up.

Recent advances, including data from the Human Microbiome Project, have lead to a new paradigm for understanding chronic, bacterially mediated diseases. Dental caries occurs as the result of a shift in the composition of a biofilm community specific to the human tooth surface^23^. S. mutans was found to be the dominant species in many, but not at all, patients with caries; S. salivarius, S. sobrinus and S. parasanguinus were also associated with caries, especially in patients with no or low levels of S. mutans^24^. Additionally the importance of microbiome community interactions in caries pathogenesis is not well understood, including the contribution of bacterial members in promoting health, such as alkali production^25^. In this retrospective observational cohort study, we analyzed data collected by the saliva sampling method, which has been widely used in Japanese general practices. This method is handy and inexpensive, but cannot identify these bacterial species. In the future study, technical advances such as 16S rRNA gene assay should be introduced for further detailed bacterial analyses.

Further studies should also include patients in other age groups not only 20 to 64 years, such as children and adolescents, who are liable to develop primary caries, and older patients, who are prone to root caries with profiles different from those of coronal caries.

In summary, survival CART analysis is an effective tool for identifying the caries risk of an individual adult patient. Cariogenic bacteria are the most influential factors contributing to the incidence of caries. Preventive programs are effective in limiting the incidence of primary carious lesion.

## Materials and Methods

### Patients

The entire database of 9,537 patients registered in nine Japanese general practices from May 1993 to February 2013 was screened. All nine clinics participating in this study shared fundamentally similar ideas regarding the diagnosis of caries, timing of operative interventions, and preventive programs. All are active in the Japan Health Care Dental Association, which promotes oral heath through preventive dental care (http://healthcare.gr.jp). In total, 1,800 patients were randomly selected, 200 from each clinic, for analysis on April 2013. We have confirmed that there were no biases in terms of the age, sex and DMFT between the selected 200 patients and the full register of patients at the nine clinics. Patients aged 20 to 64 years whose cariogenic bacteria levels and flow rate and buffer capacity of saliva had been tested when completing their initial restorative and periodontal treatments were considered eligible. Patients who met any of the following conditions were excluded: those who failed to complete the initially planned treatments; those who could not control their plaque because of physical problems; those who using an antibiotic commonly; and those who received restorative treatment in other clinics. This Study were carried out in accordance with protocols approved by the ethics committee of Osaka University Graduate School of Dentistry (E21-E28-1). All subjects were informed of the protocols and gave their written consent prior to participating in the study.

### Oral examination

The oral condition of all patients was examined by dental hygienists who had been trained and whose work was calibrated with representative cases and then double-checked by a dentist. All teeth were examined by visual inspection and bitewing x-ray. The number of teeth with caries experience (DMFT) were recorded. “D” recorded at the all clinical lesions with cavitation. Any coronal carious lesion that penetrated through one-third of the dentin was considered severe enough to require restorative treatment^26^,^27^. Secondary carious lesion referred to a substantial tooth decay at a margin of an existing restoration.

### Biological testing

Before the caries risk assessment, each patient completed initial restorative and periodontal treatment to eliminate possible negative effects of inferior oral conditions. The stimulated saliva flow rate was measured after the patients had chewed on paraffin pellets for five min. The saliva buffering capacity and levels of *mutans streptococci* (SM) and *Lactobacilli* (LB) were assessed with Dentobuff Strip, Dentocult SM Strip, and Dentocult LB kits (Orion Diagnostica, Espoo, Finland), respectively. The trained dental hygienists collected the biological samples. The dental hygienists underwent annual evaluations throughout the testing period to ensure that there was 85% agreement in evaluating the cariogenic bacteria.

### Preventive program

All patients who completed the initial restorative and periodontal treatment were advised to undergo preventive treatment against caries and periodontitis at 3–6 month intervals. The preventive treatments included education on plaque control, diet and smoking. providing scaling and polishing, and fluoride application with 9,000 ppm NaF solution. All patients used toothpaste containing 900 ppm fluoride daily; the drinking water in their residential areas was not fluoridated.

### Statistical analyses

For caries risk assessment, the following parameters for each patient were considered: age; the number of teeth with caries experience (DMFT); SM and LB levels; and saliva flow rate and buffer capacity. Additionally, each patient’s compliance with the preventive program was categorized into one of two groups: good compliance indicated regular or occasionally delayed clinic visits; and poor compliance indicated irregular or as-needed clinic visits.

Survival analysis was conducted to clarify if the preventive program was effective in reducing the incidence of carious lesion in high-risk patients. In the survival analysis, the length of time between the start of preventive therapy and the first outbreak of primary or secondary carious lesion was recorded in a period of three years. This time period was compared with the patient’s level of specific cariogenic bacteria and compliance with the preventive program. Survival analysis was conducted using the Cox hazard model, and the results were confirmed by Kaplan–Meier and log-rank tests. Data were analyzed with JMP software (version 10.0, SAS Institute, Cary, NC, USA).

Survival CART analysis was applied to develop a caries prediction model using a set of potentially significant factors. Higher and lower risks of caries were evaluated with hazard ratios using Cox proportional hazards regression analysis. Hazard ratios presented vulnerability to caries of patients in a low- or high-risk group compared to those remaining others. Data were analyzed with R software statistical package (version 3.1.0, http://www.r-statistics.com).

## Additional Information

**How to cite this article**: Arino, M. *et al*. Multicenter study on caries risk assessment in adults using survival Classification and Regression Trees. *Sci. Rep.* **6**, 29190; doi: 10.1038/srep29190 (2016).

## Supplementary Material

Supplementary Information

Supplementary Information

## Figures and Tables

**Table 1:** The result of Cox proportional hazards single regression analysis for identifying the risk of primary and secondary caries in adult patients.

Variables	Primary caries		Secondary caries	
	Hazard ratio (95% CI)	*p*-value	Hazard ratio (95% CI)	*p*-value
Age	0.99 (0.97–1.01)	0.2902	1.01 (1.00–1.03)	0.1397
DMFT	1.02 (0.98–1.06)	0.3203	1.09 (1.05–1.03)	<0.0001*
Saliva flow	0.98 (0.92–1.04)	0.5762	0.98 (0.92–1.03)	0.4261
Buffer capacity	0.84 (0.56–1.53)	0.3584	1.07 (0.78–1.44)	0.6565
SM level#	2.31 (1.41–3.99)	0.0010*	2.62 (1.70–3.98)	<0.0001*
LB level##	2.03 (1.23–3.26)	0.0027*	3.91 (2.17–7.78)	<0.0001*
Poor compliance	2.36 (1.46–3.77)	0.0006*	1.44 (0.90–2.25)	0.1229

^*^Statistically significant at a level of *p* < 0.05.

^#^Level of *mutans streptococci*. The thresholds were 1 × 10^6^ CFU/ml for primary and secondary caries.

^##^Level of *Lactobacilli*. The thresholds were 1 × 10^5^ CFU/ml and 1 × 10^5^ CFU/ml for primary and secondary caries, respectively.
