# Supplementary material for: Multicenter study on caries risk assessment in adults using survival Classification and Regression Trees
Source: Sci Rep. 2016 Jul 6;6:29190. doi: 10.1038/srep29190 (PMC4933980; doi:10.1038/srep29190)
Supplement: Supplementary Information [file srep29190-s1.pdf]

Multicenter study on caries risk assessment in adults using survival Classification and Regression Trees

Masumi Arino, Ataru Ito, Shozo Fujiki, Seiichi Sugiyama, Mikako Hayashi

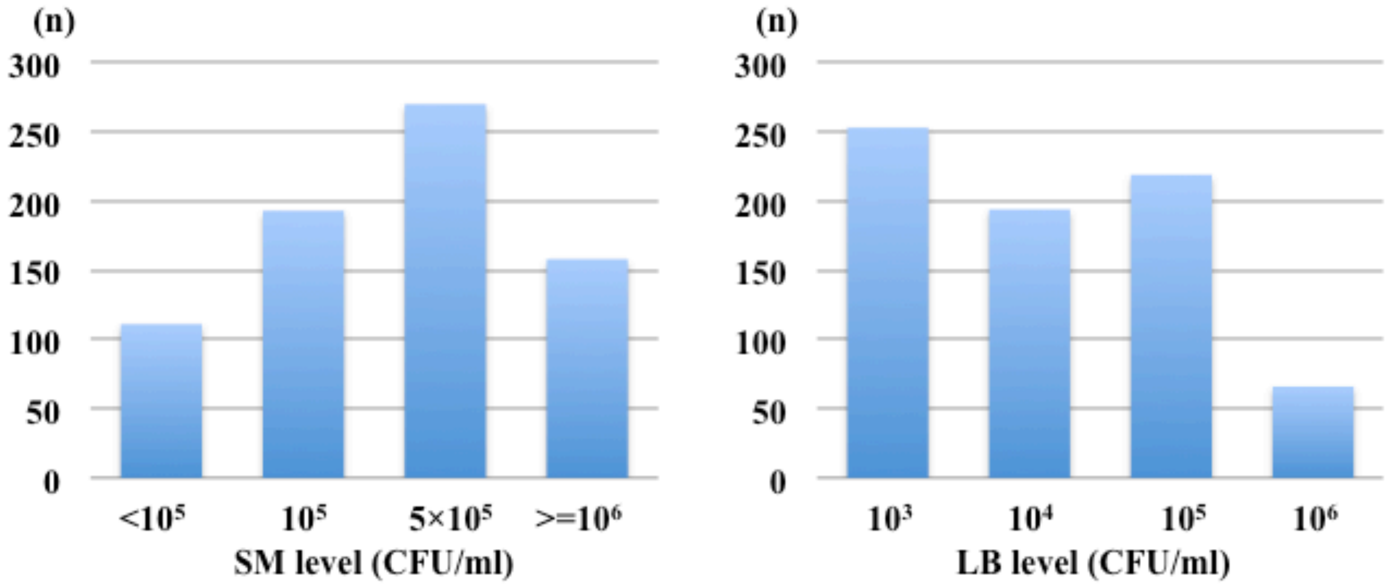

Appendix 1: Patient distribution according to levels of *mutans streptococci* (SM) and *Lactobacilli* (LB).
