# Supplementary material for: Multicenter study on caries risk assessment in adults using survival Classification and Regression Trees
Source: Sci Rep. 2016 Jul 6;6:29190. doi: 10.1038/srep29190 (PMC4933980; doi:10.1038/srep29190)
Supplement: Supplementary Information [file srep29190-s2.pdf]

Multicenter study on caries risk assessment in adults using survival Classification and Regression Trees

Masumi Arino, Ataru Ito, Shozo Fujiki, Seiichi Sugiyama, Mikako Hayashi

Appendix 2: Caries risk of patients assessed by combination of levels of cariogenic bacteria and compliance with the preventive program, by Cox hazard model.

	n	Primary caries		Secondary caries	
		**Hazard ratio (95%CI)**	***p*-value**	**Hazard ratio (95%CI)**	***p*-value**
SM: low level, Compliance: good	366	1.00 (-)	-	1.00 (-)	-
SM: high level, Compliance: good	96	1.96 (0.98-3.71)	0.0560	2.41 (1.41-4.03)	0.0017*
SM: low level, Compliance: poor	208	2.19 (1.16-4.00)	0.0160*	1.27 (0.66-2.30)	0.4646
SM: high level, Compliance: poor	62	4.73 (2.40-8.90)	<0.0001*	3.63 (1.89-6.56)	0.0003*
LB: low level, Compliance: good	168	1.00 (-)	-	1.00 (-)	-
LB: high level, Compliance: good	294	1.58 (0.30-1.23)	0.1831	4.00 (2.02-9.11)	<0.0001*
LB: low level, Compliance: poor	85	3.10 (1.18-7.77)	0.0224*	1.90 (0.41-6.69)	0.3747
LB: high level, Compliance: poor	185	3.75 (1.85-8.07)	0.0002*	6.09 (2.84-14.5)	<0.0001*

SM: high level = higher than $1x10^6$ CFU/ml, low level = $1x10^6$ CFU/ml or lower

LB: high level = higher than $1x10^4$ CFU/ml, low level = $1x10^4$ CFU/ml or lower

Each patient's compliance with the preventive program was categorized as follows:

good = regular visits and sometimes delayed visits, poor = irregular visits and emergency visits only

*Statistically significant at a level of $p < 0.05$
